# Clinical profiles of diabetic foot ulcer patients undergoing major limb amputation at a tertiary care center in North-eastern Tanzania

**DOI:** 10.1186/s12893-021-01051-3

**Published:** 2021-01-12

**Authors:** Ahmed Shabhay, Pius Horumpende, Zarina Shabhay, Andrew Mganga, Jeff Van Baal, David Msuya, Kondo Chilonga, Samwel Chugulu

**Affiliations:** 1 Department of General Surgery, Kilimanjaro Christian Medical University College, P.O. Box 2240, Moshi, Tanzania; 2 Department of General Surgery, Kilimanjaro Christian Medical Centre, P.O. Box 3010, Moshi, Tanzania; 3 Institute of Infectious Diseases and Research, Lugalo Military College of Medical Sciences (MCMS) and General Military Hospital (GMH), Mwenge area, P.O. Box 60000, Dar es Salaam, Tanzania; 4 Department of Public Health, Kilimanjaro Christian Medical University College, P.O. Box 2240, Moshi, Tanzania; 5 ZGT Academy, Hospital Group Twente, Almelo, Hengelo, The Netherlands; 6 Department of Neuro-Surgery, Muhimbili Orthopedic Institute, P.O. Box 65474, Dar es Salaam, Tanzania; 7 Kilimanjaro Clinical Research Institute (KCRI), P.O. Box 2236, Moshi, Tanzania; 8 Cardiff University, Cardiff, Wales UK

**Keywords:** Amputation, Diabetic foot ulcers, Glycemic control, Anaemia, Meggitt-wagner classification, Tanzania

## Abstract

**Background:**

Diabetic foot ulcers complications are the major cause of non-traumatic major limb amputation. We aimed at assessing the clinical profiles of diabetic foot ulcer patients undergoing major limb amputation in the Surgical Department at Kilimanjaro Christian Medical Centre (KCMC), a tertiary care hospital in North-eastern Tanzania.

**Methods:**

A cross—sectional hospital-based study was conducted from September 2018 through March 2019. Demographic data were obtained from structured questionnaires. Diabetic foot ulcers were graded according to the Meggitt-Wagner classification system. Hemoglobin and random blood glucose levels data were retrieved from patients’ files.

**Results:**

A total of 60 patients were recruited in the study. More than half (31/60; 51.67%) were amputated. Thirty-five (58.33%) were males. Fifty-nine (98.33%) had type II diabetes. Nearly two-thirds (34/60; 56.67%) had duration of diabetes for more than 5 years. The mean age was 60.06 ± 11.33 years (range 30–87). The mean haemoglobin level was 10.20 ± 2.73 g/dl and 9.84 ± 2.69 g/dl among amputees. Nearly two thirds (42/60; 70.00%) had a haemoglobin level below 12 g/dl, with more than a half (23/42; 54.76%) undergoing major limb amputation. Two thirds (23/31; 74.19%) of all patients who underwent major limb amputation had mean hemoglobin level below 12 g/dl. The mean Random Blood Glucose (MRBG) was 13.18 ± 6.17 mmol/L and 14.16 ± 6.10 mmol/L for amputees. Almost two thirds of the study population i.e., 42/60(70.00%) had poor glycemic control with random blood glucose level above 10.0 mmol/L. More than half 23/42 (54.76%) of the patients with poor glycemic control underwent some form of major limb amputation; which is nearly two thirds (23/31; 74.19%) of the total amputees. Twenty-eight (46.67%) had Meggitt-Wagner classification grade 3, of which nearly two thirds (17:60.71%) underwent major limb amputation.

**Conclusion:**

In this study, the cohort of patients suffering from diabetic foot ulcers treated in a tertiary care center in north-eastern Tanzania, the likelihood of amputation significantly correlated with the initial grade of the Meggit-Wagner ulcer classification. High blood glucose levels and anaemia seem to be also important risk factors but correlation did not reveal statistical significance.

## Background

Diabetic foot (DF) is defined as infection, ulceration, or destruction of tissues of the foot of a person with currently or previously diagnosed diabetes mellitus [1]. DF is usually accompanied by neuropathy and/or peripheral arterial disease (PAD) in the lower extremity. Diabetic foot ulcer is a foot ulcer in a person with diabetes mellitus [1] and about 8–20% of the diabetics experience a foot ulcer in their lifetime [2]. Compared to other foot ulcers, diabetic foot ulcers have 15–45% higher risk of amputation of a limb [2] and contribute to nearly 40–70% of all non-traumatic amputations [3, 4]. In Tanzania, Chalya et al. observed that nearly half (68/162; 41.9%) of the study population who underwent major limb amputation were due to complications of diabetic foot ulcers [5].

It is still not clearly understood which factors play a major role in diabetic foot ulcer patients undergoing major limb amputations [6–8] nor what role neuropathy, peripheral vascular disease, and ulcers each play in pathophysiology of major limb amputations [8]. Documented risk factors do not clearly distinguish those contributing to minor or major limb amputation [6].However peripheral neuropathy, ulceration, infection, and peripheral vascular disease [7, 9], ischaemic ulcers [6, 7] occurring early in diabetics have been identified as principal factors [6] with male sex [6, 9], size of ulcer, diabetic nephropathy [7], previous ulcer history, hypertension status, poor glycemic control [6, 7] and dyslipidemia [6] also playing a role in amputation of diabetic ulcers [4]. It should be noted that study designs, genetic profiles, ethnicity [8],state of health care system [6] and cultural characteristics might influence the discrepancies observed from different authors [7].

In patients undergoing major limb amputation, age at enrollment, male gender, type two diabetes, high body mass index, poor glycemic control, hypertension, peripheral sensory neuropathy and peripheral vascular disease were the high risk clinical profiles identified by Kantanka et al. in a study in West Africa [10]. In a study at Bugando Medical Centre, a University teaching hospital in North-western Tanzania, Chalya et al. observed Meggitt-Wagner Grade 4 ulcers patients were significantly more likely to undergo major limb amputation [11].

Major limb amputation (Transtibial/below knee and transfemoral/above knee amputation) [1, 4] is a last resort lifesaving procedure in the management of diabetic foot ulcers patients. Loss of a limb in settings where prosthetics availability is scares or financially restrictive as in Low and Middle Income countries like Tanzania leads to socio, economic and psychological effects to the surrounding patients community as a whole [5] with only half of the amputated diabetic patients having satisfactory rehabilitation [6].

Little is known on clinical profiles of the amputated diabetic foot ulcer patients in North-eastern Tanzania. We set out to determine the clinical profiles of patients who underwent major limb amputations at our centre to provide data needed for planning intervention in prevention of amputation among the diabetic foot ulcer patients.

## Materials and methods

This was a cross sectional study done in the Department of General Surgery, Kilimanjaro Christian Medical Centre, Moshi Tanzania for a period of six months from September 2018 through March 2019. A convenient sampling was done. Diabetic foot ulcer patients admitted in the Surgical department were all included during the study period.

Enrolment in the study required the patient to be above 18 years, be diagnosed with diabetes and an ulcer below the malleolus, with blood work up of haemoglobin and random blood glucose, with known information on age, duration of diabetes, type of anti-diabetic medication, previous history of amputation, hypertensive status, foot involved, duration of ulcer and an informed consent. Non-diabetic patients with foot ulcers of other etiologies were not included.

Diabetic foot ulcers were graded according to the Meggitt-Wagner classification and managed by glycemic control, daily dressings, debridement, disarticulation and amputation when necessary.

During dressing, the gauze covering the wound was irrigated with Normal saline to loosen its attachment to the underlying tissues to reduce trauma and bleeding. Upon removal of the gauzes the wound is irrigated with normal saline, minimal sloughs removed bed-side by surgical blade under local anaesthesia, gentle cleaning by normal saline-soaked wet gauze, drying of the wound with sterile dry gauze and then finally dressing up the wound. The gauzes are kept in place by zinc oxide plasters. Debridement techniques used were Surgical and sharp using scalpel. The types of amputation were either transtibial or transfemoral.

### Data management and statistical analysis

Data were collected using a structured questionnaire. Categorical data were expressed as proportions or percentages. Meggitt-Wagner Classification ulcer grading and outcomes of debridement, disarticulation or amputation were determined from clinical notes/post-operative notes upon discharge. Comparisons between proportions were done using Chi square or Fischer’s exact test. A p value of less than or equal to 0.05 at 95% confidence interval was considered statistically significant.

### Ethical considerations

The study proposal was submitted, reviewed and approved by the Kilimanjaro Christian Medical University College Research and Ethical Committee (CREC) and granted certificate number 2366.

The study was conducted according to the ethical principles for medical research in accordance to the Declaration of Helsinki. Confidentiality was ensured in that no personal identifying information was written in the data capture or database. No individual person’s data in any form (including any individual details, images or videos) were included. Written informed consent to participate in the study was obtained from study participants. Participants were clearly made to understand that not participating in the study would in no way jeopardize clinical management in the ward.

## Results

### Demographic characteristics

A total of 60 patients were recruited in the study. Thirty-five (58.33%) were males. The mean age was 60.06 ± 11.33 years (range 30–87) Table 1.

**Table 1:** Demographic characteristics

Variable	n (%)	Amputation	Debridement	Disarticulation	*P*-value
		n (%)	n (%)	n (%)	
Age in years (mean(SD))	60.06 (11.33)	58.03 (8.33)	61.16 (13.34)	65.50 (14.94)	n/a
Age in years					
≤ 40 years	1 (1.67)	0 (0.00)	1 (100.00)	0 (0.00)	0.416
41–50 years	10 (16.67)	5 (50.00)	4 (40.00)	1 (10.00)	
51–60 years	24 (40)	16 (66.67)	6 (25.00)	2 (8.33)	
> 60 years	25 (41.67)	10 (40.00)	10 (40.00)	5 (20.00)	
Sex					
Female	25 (41.67)	13 (52.00)	9 (36.00)	3 (12.00)	> 0.999
Male	35 (58.33)	18 (51.43)	12 (34.29)	5 (14.29)	
Total	60 (100.00)	31 (51.67)	21 (35.00)	8 (13.33)	

### Clinical characteristics

The mean haemoglobin level was 10.20 ± 2.73 and 9.84 ± 2.69 g/dl for patients undergoing major limb amputation. The mean random blood glucose was 13.18 ± 6.17 mmol/L and 14.16 ± 6.10 mmol/L for amputees Table 2. Fifty-nine (98.33%) patients were diagnosed with diabetes type II. Nearly two-thirds 34(56.67%) had duration of diabetes for more than 5 years. More than half (31/60; 51.67%) of the patients were amputated, nearly a quarter (21/60; 35.005) needed debridement with only (8/60; 13.33%) undergoing disarticulation of digits. Nearly two thirds (42/60; 70.00%) had a haemoglobin level below 12 g/dl, with more than a half (23/42; 54.76%) undergoing major limb amputation. This is almost two thirds (23/31; 74.19%) of all patients who underwent major limb amputation. Almost two thirds i.e., 42/60 (70.00%) had poor glycemic control with random blood glucose level above 10.0 mmol/L of which more than half 23/42 (54.76%) underwent some form of major limb amputation which is nearly two thirds (23/31; 74.19%) of the total amputees. Thirty-seven (61.67%) were not on insulin therapy where more than half (20/37; 54.05%) underwent major limb amputation, this is nearly two thirds (20/31; 64.52%) of the total amputees. Fifty-seven (95.00%) had no prior history of amputation. Almost half, 28 (46.67%) had Meggit-Wagner classification grade 3, of which nearly two thirds (17:60.71%) underwent major limb amputation, nearly a quarter (13; 21.67%) had Meggit-Wagner grade 4 and 1 (1.67%) had Meggitt-Wagner grade 5, with 100% amputation rate. A quarter 16 (26.67%) had Meggitt-Wagner grade 2 and 2 (3.33%) had Meggitt-Wagner grade 1 with no a mputations. (*P* < 0.001) which is statistically significant Table 3.

**Table 2:** Means of continuous variables versus outcome

Variable	Overall	Amputation	Debridement	Disarticulation
	Mean(± SD)	Mean(± SD)	Mean(± SD)	Mean(± SD)
Age in years	59.75(11.45)	58.19(8.41)	59.86(13.61)	65.50(14.94)
Hemoglobin (g/dl)	10.20(2.73)	9.84(2.69)	10.48(2.92)	10.88(2.43)
RBG (mmol/l)	13.81(6.17)	14.16(6.10)	14.16(6.50)	11.53(5.81)

**Table 3:** Clinical characteristics versus outcomes

Variable	n (%)	Amputation	Debridement	§Disarticulation	*P*-value
		n (%)	n (%)	n (%)	
DM type					
Type I	1 (1.67)	0 (0.00)	1 (100.00)	0 (0.00)	0.483
Type II	59 (98.33)	31 (52.54)	20 (33.90)	8 (13.56)	
Duration of DM in years					
< 1	5 (8.33)	1 (20.00)	2 (40.00)	2 (40.00)	0.118
1–5	21 (35.00)	12 (57.14)	5 (23.81)	4 (19.05)	
> 5	34 (56.67)	18 (52.94)	14 (41.18)	2 (5.88)	
Antidiabetic agent					
Injection	23 (38.33)	11 (47.83)	10 (43.48)	2 (8.70)	0.66
Oral	32 (53.33)	18 (56.25)	9 (28.13)	5 (15.63)	
Herbal	5 (8.33)	2 (40.00)	2 (40.00)	1 (20.00)	
Prior history of amputation					
No	57 (95.00)	30 (52.63)	19 (33.33)	8 (14.04)	0.715
Yes	3 (5.00)	1 (33.33)	2 (66.67)	0 (0.00)	
Meggitt-Wagner classification					
1	2 (3.33)	0 (0.00)	2 (100.00)	0 (0.00)	< 0.001
2	16 (26.67)	0 (0.00)	10 (62.50)	6 (37.50)	
3	28 (46.67)	17 (60.71)	9 (32.14)	2 (7.14)	
4	13 (21.67)	13 (100.00)	0 (0.00)	0 (0.00)	
5	1 (1.67)	1 (100.00)	0 (0.00)	0 (0.00)	
Haemoglobin (g/dl)					
< 7	8 (13.33)	4 (50.00)	3 (37.50)	1 (12.50)	0.525
07–10	23 (38.33)	15 (65.22)	7 (30.43)	1 (4.35)	
> 10–12	11 (18.33)	4 (36.36)	4 (36.36)	3 (27.27)	
> 12	18 (30.00)	8 (44.44)	7 (38.89)	3 (16.67)	
RBG (mmol/l))					
< 10	18 (30.00)	8 (44.44)	7 (38.89)	3 (16.67)	0.94
10–19	28 (46.67)	15 (53.57)	9 (32.14)	4 (14.29)	
≥ 20	14 (23.33)	8 (57.14)	5 (35.71)	1 (7.14)	
Hypertensive					
No	34 (56.67)	18 (52.94)	11 (32.35)	5 (14.71)	0.877
Yes	26 (43.33)	13 (50.00)	10 (38.46)	3 (11.54)	
duration of ulcers (months)					
< 1	40 (66.67)	22 (55.00)	13 (32.50)	5 (12.50)	0.744
≥ 1	20 (33.33)	9 (45.00)	8 (40.00)	3 (15.00)	
Total	60 (100.0)	31 (51.67)	21 (35.00)	8 (13.33)	

§ loss of foot digits or part of it

## Discussion

We set out to describe the clinical profiles of diabetic foot ulcer patients who underwent major limb amputations as part of their clinical management admitted in the surgical department of KCMC, a tertiary care and a University teaching hospital in north-eastern Tanzania. It is important to identify factors contributing to amputation among diabetic foot ulcers patients for intervention. In this study population anemia (23/31; 74.19%), poor glycemic control (23/31;74.19%) and Meggit-Wagner’s ulcer grade 3 and above (42/60; 70.00%) were risk factors for major limb amputation.

In our study 18/60(30.00%) patients had random blood glucose of less than 10.0 mmol/L on admission. Almost two thirds i.e., 42/60(70.00%) of the study population had poor glycemic control medically with random blood glucose level above 10.0 mmol/L Table 3. This is a high blood glucose level that may explain why these patients developed diabetic ulcers. The recommended glycemic targets in non-pregnant diabetics are a pre-prandial capillary plasma glucose of 80–130 mg/dL (4.4–7.2 mmol/L) or peak postprandial capillary plasma glucose < 180 mg/dL (10.0 mmol/L) [12]. Of the 42/60 (70.00%) patients with poor glycemic control, more than half 23/42(54.76%) underwent some form of major limb amputation. A total of 31 patients underwent some form of major limb amputation. Poor glycemic control contributed to nearly two quarters (23/31; 74.19%) of the total amputees. In the diabetic population chronic hyperglycemia leads to impaired wound healing due to increased susceptibility to infections, chronic inflammation state, diabetic micro and macro-angiopathy leading to diminished vascularity, impaired collagen synthesis, impaired hyaluronan, autonomic dysfunction and abnormality in cell-mediated immunity and phagocytic function [13, 14]. A high glycated hemoglobin also impairs endothelium mediated vasoactive responses. An erythrocyte shape is affected by a high glucose level, making blood more viscous, impeding blood flow and facilitating formation of thrombus thereby increasing the risk of amputation [15]. Thus, Blood glucose control is paramount in healing of ulcers in diabetics [16].

Whilst tight glycemic control is paramount to prevention of both short- and long-term diabetic complications [6] our patients had a poor glycemic control. More than half 32/60 (53.33%) were on oral hypoglycemics but had a poor glycemic control. Patients on oral hypoglycemics alone and with a poor control require insulin for adequate control of the blood glucose [17]. Only a quarter (23/60; 38.33%) of our patients were on injectable insulin at the time of presentation. Of the 37/60 (64.52%) patients who were not on insulin therapy, more than half (20/37; 54.05%) underwent major limb amputation, this is nearly two thirds (20/31; 64.52%) of the total amputees. We opine that these patients were medically mismanaged and ended in surgery. It has been observed that male diabetic foot ulcer patients not on insulin therapy have increased risk of undergoing a major limb amputation. However a clear cut information on the indications for initiating insulin in diabetic foot ulcers have to be determined [18].

Thus, an effective regimen on the optimal management of a patient has to be individualized depending on their comorbid conditions [12]. The choice of anti-diabetic medication in diabetic foot ulcer patients depends on the severity of infection. However, in a limb threatening foot ulcer, insulin must be initiated [18]. Tight glycemic control is paramount in infection eradication and ulcer healing [6].

In this study, the mean Haemoglobin level was 10.20 ± 2.73 and 9.84 ± 2.69 g/dl for patients undergoing major limb amputation. Nearly two thirds of our patients (42/60; 70.00%) had a haemoglobin level below 12 g/dl, more than a half of these anemic (23/42; 54.76%) undergoing major limb amputation. This is almost two thirds (23/31; 74.19%) of all patients who underwent major limb amputation in our study showing that anaemia was an important factor for amputation Table 3. The World Health Organization cut off point for diagnosis of anaemia is a hemoglobin level of 12.0 g/dl for females and 13.0 g/dl for males [19, 20]. Diabetics have a twofold chance of developing anaemia [20–23]. In anemic diabetic foot ulcers patients a poor prognosis of the ulcer healing is evident [21]. Costa et al. had a similar finding where 89.6% of patients who underwent major limb amputation were anaemic, and anaemia was a significant risk factor for major limb amputation [20, 24]. There is more evidence for a strong association between anaemia and amputation [21]. In diabetic patients with peripheral arterial disease, anaemia further exaggerates the effects of tissue hypoperfusion due to poor oxygen delivery to peripheral tissues and thrombus formation [21].

About half of our patients, 28/60 (46.67%) had Meggit-Wagner classification grade 3 of which nearly two thirds (17/28; 60.71%) underwent major limb amputation. About a quarter (13/60; 21.67%) had Meggit-Wagner grade 4 and one (1/60; 1.67%) grade 5 of which all underwent major limb amputation Table 3. The Meggitt-Wagner classification system historically is the most frequently used classification system of diabetic foot ulcer [3, 25, 26]. It is a six grade classification system described by Meggitt in 1976 and disseminated by Wagner in 1979 [1] Table 4. In this classification grade 0–3 is mainly based on neuropathy with grade 4–5 representing ischaemic lesions [25].It is based on wound depth and tissue viability [26] and assess presence of osteomyelitis [27]. The Meggitt-Wagner classification system grades pre-ulcerative lesions (grade 0); superficial ulcer (grade 1); deep ulcer involving tendon (grade 2); ulcer with bone involvement or abscess (grade 3); limited foot gangrene (grade 4) and whole foot gangrene (grade 5) [27]. It was used in this study due to its predictive value of treatment outcome. It has been observed that Meggit-Wagner grading of diabetic foot ulcer affects and predicts the outcome and the increased risk of amputation [25, 27]. In our study, nearly two thirds of grade 3 ulcer patients and all patients with grade 4 and 5 ulcers underwent major limb amputation. Despite its limitations of not taking into consideration the loss of protective sensation, its inability to differentiate infected and/or ischemic ulcers, with some authors describing the system as imprecise, it has been documented to be able to show association between grade and loss of limb [26]. In our study the Meggitt-Wagner classification system showed an increased amputation risk with higher grades with statistically significant results. Our data give further evidence of the utility and usefulness of the Meggitt-Wagner classification system in predicting major limb amputation among diabetic ulcer patients.

**Table 4:** Meggit-Wagner ulcer classification system [3]

Grade	Description
0	Pre- or post-ulcerative lesion completely epithelialized
1	Superficial, full-thickness ulcer confined to the dermis, not extending to the subcutaneous tissues
2	Ulcer of the skin extending through the subcutaneous tissues with exposed tendon or bone and without osteomyelitis or abscess formation
3	Deep ulcers with osteomyelitis or abscess formation
4	Localized gangrene of the toes or the forefoot
5	Foot with extensive gangrene

This study had several limitations. It was time bound and focused only on the objectives set. On the surgical management of diabetic foot ulcers, information on microbiological profile of micro-organisms isolated on infected ulcers with their antibiotic sensitivity profile results, presence of osteomyelitis (clinical and radiological), duration of conservative treatment, the mean length of hospital stay, number of previous debridement’s prior major limb amputation, indication for amputation, level of amputation, techniques of amputation, and prosthesis supply status is of paramount importance. A study on diabetics, its co-morbidities and its surgical complications is wide and vast, thus in this study, due time and resource limitations, we could only address some risk factors, namely hypertension, Meggitt-Wagner ulcer grade status of patients, haemoglobin levels and blood sugar levels and their association with lower limb amputation. With regards to prosthesis supply, there is an Orthotics and prosthetic department in KCMC hospital, where diabetic foot ulcer patients post amputation after stump healing are referred for prosthesis fitting. A big challenge in our centre is follow-up. Our center caters as a specialized referral hospital for the northern zone of Tanzania. Most of these patients reside from distant localities. Once amputated, they return back to their locality and feedback from them is a challenge. Few return for follow up clinics and prosthesis fitting as it is cost restrictive.

## Conclusion

In this study, the cohort of patients suffering from diabetic foot ulcers treated in a tertiary care center in north-eastern Tanzania, the likelihood of amputation significantly correlated with the initial grade of the Meggit-Wagner ulcer classification. High blood glucose levels and anaemia seem to be also important risk factors but correlation did not reveal statistical significance.

## Data Availability

The data used to support this study are available from the first author upon request.

## Abbreviations

HbA1c: Glycosylated Hemoglobin
KCMC: Kilimanjaro Christian Medical Centre
MRBG: Mean Random Blood Glucose
PAD: Peripheral Arterial Disease
